# The Biological Function and Therapeutic Potential of Exosomes in Cancer: Exosomes as Efficient Nanocommunicators for Cancer Therapy

**DOI:** 10.3390/ijms21197363

**Published:** 2020-10-05

**Authors:** Jeong Uk Choi, In-Kyu Park, Yong-Kyu Lee, Seung Rim Hwang

**Affiliations:** 1 College of Pharmacy, Chonnam National University, 77 Yongbong-ro, Buk-gu, Gwangju 61186, Korea; cju0667@jnu.ac.kr; 2 Department of Biomedical Sciences, Chonnam National University Medical School, 322 Seoyang-ro, Hwasun 58128, Korea; pik96@jnu.ac.kr; 3 Department of Chemical and Biological Engineering, Korea National University of Transportation, 50 Daehak-ro, Chungju, Chungbuk 27469, Korea; leeyk@ut.ac.kr; 4 College of Pharmacy, Chosun University, 309 Pilmun-daero, Dong-gu, Gwangju 61452, Korea; 5 Department of Biomedical Sciences, Graduate School, Chosun University, 309 Pilmun-daero, Dong-gu, Gwangju 61452, Korea

**Keywords:** exosome, cancer, nanocommunicator, diagnostic biomarker, drug delivery vehicle, personalized cancer immunotherapy

## Abstract

Cancer therapeutics must be delivered to their targets for improving efficacy and reducing toxicity, though they encounter physiological barriers in the tumor microenvironment. They also face limitations associated with genetic instability and dynamic changes of surface proteins in cancer cells. Nanosized exosomes generated from the endosomal compartment, however, transfer their cargo to the recipient cells and mediate the intercellular communication, which affects malignancy progression, tumor immunity, and chemoresistance. In this review, we give an overview of exosomes’ biological aspects and therapeutic potential as diagnostic biomarkers and drug delivery vehicles for oncotherapy. Furthermore, we discuss whether exosomes could contribute to personalized cancer immunotherapy drug design as efficient nanocommunicators.

## 1. Introduction

Oncology drugs constitute the largest therapeutics section approved by the Center for Drug Evaluation and Research, a division of the United States Food and Drug Administration [1]. Once cancer metastasizes from the primary tumor to new sites at the time of detection, the survival rate of cancer patients decreases substantially, posing a threat to overall health [2]. In recent times, an early diagnosis of cancer via timely screening using liquid biopsy tools such as circulating tumor cells (CTCs) or circulating tumor DNA (ctDNA) as well as extracellular vesicles (EVs) has received attention, and the survival rate of cancer patients has increased with the development of treatment strategies [3,4]. 

Anticancer drug approval trends have changed since cancer chemotherapeutic agents were first developed in the 1940s. Cancer chemotherapeutics show efficacy as well as side effects as they not only interfere with the growth or division of cancer cells but also normal cells. Cancer cells even evade anticancer drugs by mediating a cellular efflux of drugs or by reducing target gene expression. They not only receive external signals but also transmit signals to form new blood vessels in cancer tissues. EVs secreted by cancer cells transfer signals for cancer progression and metastasis [5].

As technical limitations associated with cancer chemotherapy have been recognized, and the understanding of cancer biology has improved, the paradigm of research and development has shifted towards targeted therapy using monoclonal antibodies and small molecules that target the signaling process of cancer cells. Recently, synthetic small molecules such as a FGFR (fibroblast growth factor receptor) inhibitor (erdafitinib; Janssen Pharmaceutica, Beerse, Belgium), CSF1R (colony-stimulating factor-1 receptor)/RTK (receptor tyrosine kinase)/FLT3 (FMS-like tyrosine kinase 3) inhibitor (pexidartinib; Daiichi Sankyo, Tokyo, Japan), and exportin 1 inhibitor (selinexor; Karyopharm Therapeutics, Newton, MA, USA), and biologics such as an antibody-drug conjugate (ADC) targeting nectin-4 (enfortumab vedotin; Astellas Pharma, Tokyo, Japan), and ADC targeting CD79B (polatuzumab vedotin; Genentech/Roche, South San Francisco, CA, USA) have been approved. Interestingly, entrectinib (Genentech/Roche) simultaneously targets c-ROS oncogene 1 (ROS1), ALK (anaplastic lymphoma kinase) RTKs, and tropomyosin receptor kinase proteins encoded by neurotrophic tyrosine receptor kinase (NTRK) genes; it was approved as a biomarker-based treatment for ROS1-positive and NTRK fusion-positive cancer. In the same vein, engineered EVs that carry small interfering RNA (siRNA) or short hairpin RNA specifically targeting oncogenic KRAS mutation showed their therapeutic potential in pancreatic ductal adenocarcinoma mouse models [6].

Despite the remarkable performance of targeted anticancer drugs, limitations have been associated with the targeting of markers on the cancer cell surface, because cancer cells are genetically unstable, and surface proteins in cancer cells change dynamically during disease progression [7]. The necessity of developing new therapeutic approaches has emerged due to the genetic heterogeneity between cancer cells and drug resistance mechanisms [8]. Since 2010, cancer immunotherapy drugs including CTLA4 (cytotoxic T-lymphocyte-associated protein 4) inhibitors, PD-1 (programmed cell death protein 1) inhibitors, PD-L1 (programmed cell death ligand 1) inhibitors, and CAR (chimeric antigen receptor)-T cell therapy have received a lot of attention. As cancer cells proliferate by evading the immune system, cancer immunotherapy drugs interfere with the evasion mechanism or stimulate immune cells to attack tumor cells [9]. 

However, cancer therapeutics encounter barriers against transport to target sites owing to the elevated levels of solid stress, vascular network formation, interstitial fluid pressure, and density of extracellular matrix (ECM) in the tumor microenvironment [10]. Nanocarriers can enhance the permeability and retention of their cargo drugs in solid tumor tissues. Certain cancer therapeutics need to be delivered to intracellular targets such as the cytosol or nucleus to elicit their proper action [11]. Interestingly, nanosized EVs called exosomes, transfer their cargo nucleic acids and proteins to the recipient cells via the cellular uptake of vesicles; this contributes to the intercellular communication between tumor cells and bone marrow stromal cells. Recurrent mutations or specific alterations of niches within hematopoietic cells of the bone marrow regulating the production of blood and immune cells play roles in malignancy progression and chemoresistance [12]. In that exosomes orchestrate immune cells in the tumor microenvironment through cell-to-cell signaling, they have been tested for cancer immunotherapy in clinical trials. Dendritic cell (DC)-derived exosomes (DEXs) showed modest efficacy in patients with metastatic melanoma and non-small cell lung cancer (NSCLC) [13,14]. Autologous tumor-derived exosomes (TEXs) in combination with the GM-CSF (granulocyte-macrophage colony-stimulating factor) could induce antitumor T lymphocyte response in colorectal cancer [15]. TEXs can also provide diagnostic biomarkers, because they circulate in biological fluids and the exosomal components enclosed in lipid membrane vesicles reflect the characteristics of the cells of origin in the tumor tissue. 

In this review, we aimed to provide a comprehensive overview of the biological aspects and potential therapeutic applications of exosomes in cancer. Here, we also discuss whether exosomes could contribute to personalized cancer immunotherapy drug design as efficient nanocommunicators.

## 2. Biologic Aspects of Exosomes and Cancer

### 2.1. Exosome Biogenesis

**Figure 1:**
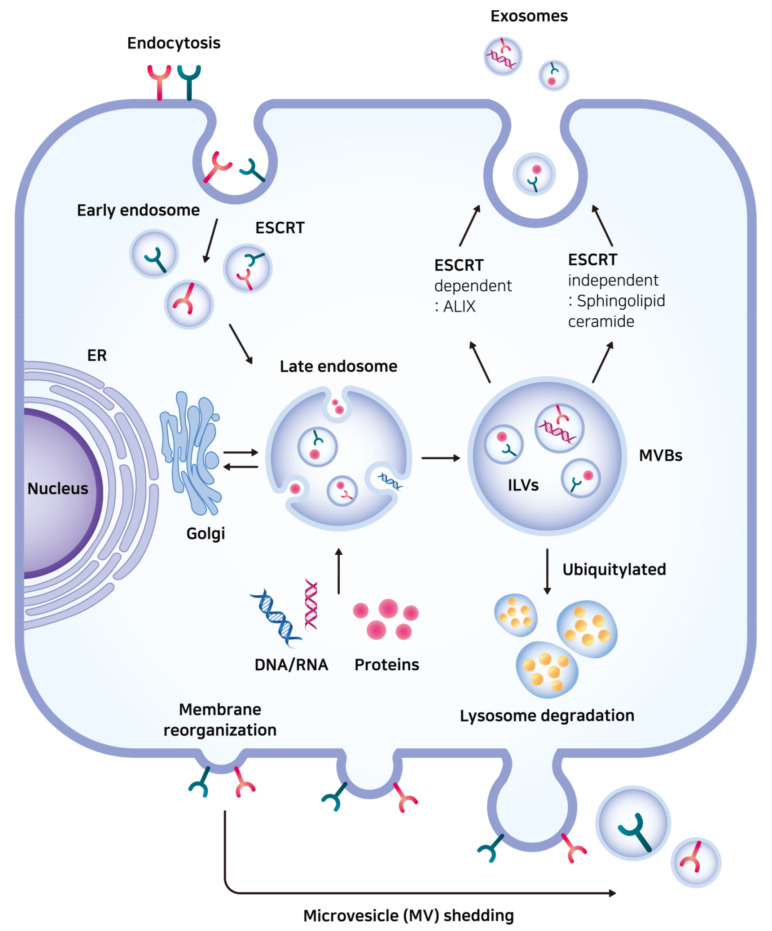
The process of exosome biogenesis. The perimeter membrane of endocytic vesicles buds inward during endosome maturation from the early endosome to the late endosome. Further invagination of the endosomal membrane forms intraluminal vesicles (ILVs) in the multivesicular body (MVB). Subsequently, the MVB is fused with the lysosome or release its contents in the form of exosomes (top right). This process of exosome biogenesis is different from that of microvesicle (MV) shedding (bottom). Receptors internalized from the cell surface and other exosomal cargo proteins are packed in the late endosome either by endosomal sorting complex required for transport (ESCRT)-dependent or ESCRT-independent pathway. ER: endoplasmic reticulum; ILV: intraluminal vesicle; MVB: multivesicular body; MV: microvesicle; ESCRT: endosomal sorting complex required for transport; ALIX: apoptosis-linked gene 2-interacting protein X.

Cells release EVs enclosed in lipid membranes into the extracellular environment. Exosomes, microvesicles (MVs)/microparticles, and apoptotic bodies form a subgroup of EVs. They have been defined by their biogenesis, size, or constituent molecules. The process of exosome biogenesis starts with endocytic membrane transport through which the cell surface proteins can be recycled [16]. The perimeter membrane of endocytic vesicles buds inward during endosome maturation from the early endosome to the late endosome [17]. Further invagination of the endosomal membrane into the endosomal compartment forms intraluminal vesicles (ILVs) in the multivesicular body (MVB). Subsequently, the MVB is either fused with the lysosome for degradation or release its contents in the form of exosomes by merging with the plasma membrane [18]. This process of exosome formation is different from that of MV/microparticle formation that takes place via outward budding directly from the plasma membrane (Figure 1) [18].

The most well-known mechanism for packaging receptors internalized from the cell surface and other exosomal cargo proteins in the late endosome membrane depend on the endosomal sorting complex required for transport (ESCRT) machinery [19]. Cytosolic protein complexes composed of ESCRT-0, ESCRT-I, ESCRT-II, and ESCRT–III, together with accessory proteins, participate in binding ubiquitinated cargo and sculpting MVB vesicles [20]. The addition of a regulatory ubiquitin protein to the substrate is a reversible post-translational modification catalyzed by a ubiquitin-activating enzyme, ubiquitin-conjugating enzyme, and ubiquitin ligase [21]. Even though it is debatable whether the contents of MVBs are released into the extracellular medium or enter the lysosome under certain circumstances, the tagging of misfolded or damaged proteins with ubiquitin plays a role in maintaining intracellular protein levels for cell cycle regulation and is also associated with the oncogenic processes [22]. For example, mutations in genes encoding components for ubiquitin ligase activity lead to the development of renal cell carcinoma and breast cancer [23,24]. 

Alternatively, ESCRT-independent pathways are supported by MVB formation even in the depletion of key subunits of ESCRTs [25]. Sphingolipids and cholesterol that are enriched in detergent-resistant membrane domains may be involved in ubiquitin-independent protein sorting [26]. The sphingolipid ceramide also triggers the formation of ILVs in the late endosome that are destined for secretion as exosomes [27]. Observation of human lymphoblastoid cells via immunoelectron microscopy demonstrated low cholesterol labeling in the lysosome but high cholesterol labeling in the MVB and exosomes [28]. Despite the possibility of ubiquitin-independent exosomal cargo sorting, certain ESCRT components are involved in exosome formation. Apoptosis-linked gene 2-interacting protein X (ALIX), an ESCRT accessory protein, contributes to the sorting of transferrin receptor into the late endosome membrane and interacts with syntenin-linking syndecan-mediated signaling [29,30]. Hepatocyte growth factor-regulated tyrosine kinase substrate (HRS), an ESCRT-0 protein, is related to exosomal secretion and antigen-presenting activity in DCs [31]. 

The pathways of packaging RNAs into exosomes are still unclear. Specific linear sequence motifs that are shared by exosomal RNAs may function as *cis*-acting elements that target RNAs to exosomes [32]. GW-bodies containing protein components of RNA-induced silencing complex congregate with the endosome and MVB, where microRNA (miRNAs) are enriched. The exosome-like vesicles secreted by MVBs are rich in GW182, which modulates miRNA loading or gene silencing [33]. 

### 2.2. Exosome–Cell Interaction and Biodistribution of Exosomes

After being secreted by the original cell into the extracellular space, exosomes circulate in body fluids or are distributed into the tissue ECM [34,35]. Owing to their nanosize, they even penetrate the nasal mucosa and bypass the blood-brain barrier [36]. Labeling and tracking exosomes using fluorescence or bioluminescence helps us understand exosome–cell interaction and the biodistribution of exosomes [37]. 

**Figure 2:**
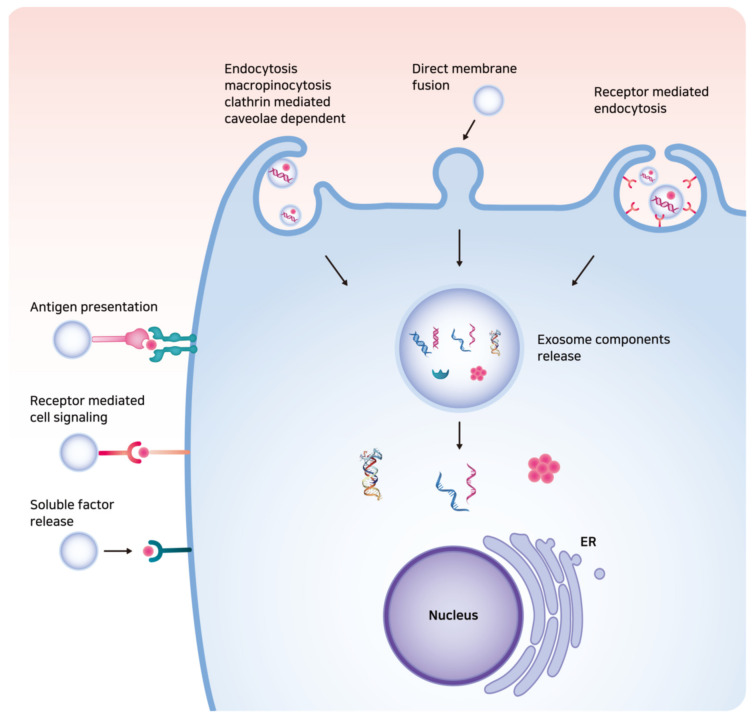
The illustration of exosome–cell interaction. Exosomes can be taken up into the recipient cell via direct membrane fusion or endocytosis, leading to the delivery of exosomal contents such as DNAs, messenger RNAs, long non-coding RNAs, enzymes, and signaling peptides or proteins to the cytosolic space of the recipient cell. Receptor-ligand interactions between cell surface receptors and exosomal ligands may also occur.

Exosomes with lipid bilayer structures can be taken up into the recipient cell via membrane fusion, clathrin-mediated endocytosis, caveolin-dependent endocytosis, macropinocytosis, or phagocytosis, leading to the delivery of exosomal contents to the cytosolic space of the recipient cell (Figure 2) [38,39,40,41]. Exosomal cargo is then released by the acidification of the endo/lysosome compartment in the recipient cell [42]. Receptor-ligand interactions between cell surface receptors and exosomal ligands may also occur based on specific cell types, and mediate antigen presentation, cell signaling, the release of soluble factor, disease progression, and immune surveillance [43]. 

**Figure 3:**
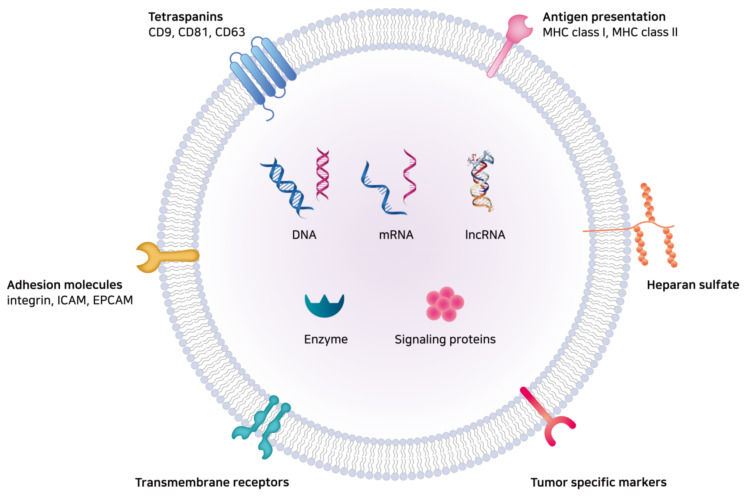
The illustration of exosomal components. The diverse functions of exosomes are governed by the delivery of exosomal cargo proteins and nucleic acids to the recipient cells. Exosomal components include lipids, nucleic acids, tetraspanins, adhesion molecules, antigen-presenting molecules, transmembrane receptors, MVB formation proteins, membrane trafficking proteins, enzymes, signaling proteins, etc. mRNA: messenger RNAs; lncRNA: long non-coding RNA; ICAM: intercellular adhesion molecule; EpCAM: epithelial cell adhesion molecule; MHC: major histocompatibility complex.

The diverse functions of exosomes are governed by the delivery of exosomal components, including lipids, nucleic acids, and proteins such as tetraspanins, adhesion molecules, antigen-presenting molecules, transmembrane receptors, MVB formation proteins, membrane trafficking proteins, cytoskeletal proteins, enzymes, signaling proteins, and heat shock proteins (Figure 3) [44]. Finally, clearance of exosomes from the body might take place via the liver, spleen, and kidneys with the mononuclear phagocytic system [45].

### 2.3. The Biological Functions of Exosomes in Cancer

Exosomes function as unique intercellular communicators and debris managers for cellular homeostasis [46]. Delivery of exosomal cargo mediates cell motility, immune responses, and reprogramming of the tumor microenvironment. Whether exosomes promote cancer progression and escape from immunosurveillance depends on the type of the cells of origin and malignancy at the time of exosome release [47,48]. TEXs have autocrine and paracrine roles in cancer progression [49]. At the site of the primary lesion, they carry fibronectin and proteinases, including membrane type 1-matrix metalloproteinase (MMP) and MMP2, and facilitate adhesion and invasiveness of cancer cells [50]. Delivery of miRNAs via TEXs transforms fibroblasts into cancer-associated fibroblasts (CAFs) [51,52,53]. Meanwhile, fibroblast-derived exosomes have been reported to stimulate directional movements of breast cancer cells, which is dependent on Wnt-planar cell polarity signaling [54]. Exosomes secreted from CAFs are rich in disintegrin and metalloproteinase domain-containing protein 10, which can enhance cancer cell motility via Notch receptor activation and the GTPase RhoA signalling [55]. Adipocyte-derived exosomes have been shown to increase migration and invasion of melanoma cells via fatty acid oxidation [56].

Exosomes also regulate angiogenesis and vascular permeability [57]. A mucin-type podoplanin glycoprotein, which is upregulated in certain types of cancer and incorporated into exosomes, reprograms exosomal proteins and promotes lymphangiogenesis [58]. Uptake of leukemia-derived exosomes containing miR-92a by endothelial cells enhanced endothelial tube formation [59]. Exosomes released by metastatic tumor cells led to endothelial hyperpermeability contrary to the exosomes released by non-metastatic tumor cells [60]. Under hypoxia, miR-23a upregulation in TEXs leads to the accumulation of hypoxia-inducible factor-1 α, enhancing angiogenesis, and inhibits tight junction protein ZO-1, increasing vascular permeability [61]. 

After the intravasation of tumor cells, TEXs traveling through the bloodstream develop “pre-metastatic niches” by modifying microenvironments in distant target organs and affecting organ-specific stromal cells [62]. Exosomes from highly metastatic melanomas reprogrammed bone marrow progenitor cells, resulting in exosome-mediated tyrosine-protein kinase Met signaling [63]. Uptake of exosomes derived from pancreatic ductal adenocarcinomas by Kupffer cells induced transforming growth factor-β secretion and fibronectin production, which initiated liver pre-metastatic niche formation [64]. Specific integrin expression patterns on TEXs were shown to correlate with the localization of TEXs and organotropic metastasis [65]. Targeting the exosomal integrin α_6_β_4_ was associated with lung metastasis, whereas targeting the exosomal integrin α_v_β_5_ was associated with liver metastasis. TEXs can also enter sentinel lymph nodes and influence lymph node distribution of cancer cells, which is driven by synchronized molecular signals that affect tumor metastasis [66]. Meanwhile, reports indicate that exosomes from non-metastatic cells inhibit metastasis. Exosomes isolated from non-metastatic patient sera suppressed experimental lung metastasis by increasing the number of patrolling monocytes in the lungs and inducing macrophage differentiation, leading to immune surveillance in the pre-metastatic niche [67].

As TEXs contain immunosuppressive ligands as well as immunostimulatory tumor-associated antigens (TAAs), they can play roles in mediating tumor immunity [68]. Binding immune-inhibitory ligands of TEXs to T cell receptors and IL-2 receptors leads to tolerogenic signals [69]. TEXs also induce apoptosis of CD8^+^ T lymphocytes and differentiation of myeloid precursor cells and regulatory T cells. They can also inhibit the cytotoxic functions of natural killer (NK) cells via downregulation of NK group 2D, an NK-activating receptor that recognizes ligands on the surface of malignant cells as well as TEXs [70]. Meanwhile, DEXs pulsed with tumor peptides activate cytotoxic T lymphocytes [71]. Mast cell-derived exosomes associated with antigens induce maturation of DCs. Antigen presentation by DCs activates B and T cells [72]. 

Exosomes have also been reported to mediate cancer chemoresistance by cargo transfer [73]. Exosomes derived from drug-resistant breast cancer cells modulated the cell cycle and drug-induced apoptosis, which might be dependent on selective miRNA patterns [73]. Restoration of miR-151a via exosomes derived from temozolomide (TMZ)-resistant glioblastoma multiforme enhances chemosensitivity to TMZ, whereas miR-151a loss drives TMZ resistance [74]. In cisplatin-resistant tumor cells, acidic pH in the extracellular microenvironment reduces cisplatin uptake into tumor cells and increases cisplatin levels eliminated via TEXs [75]. Exosomes isolated from fibroblast-derived conditioned medium prime cancer stem cells and promote chemoresistance in colorectal cancer [76]. Transfer of exosomal RNA from stromal to breast cancer cells activates signal transducer and activator of transcription 1 and NOTCH3 signaling, which regulate the expansion of chemoresistant cancer cells [77]. 

## 3. Potential Therapeutic Applications of Exosomes in Cancer

### 3.1. Exosomes as Diagnostic Biomarkers for Cancer

#### 3.1.1. Identification Techniques of Exosomes in Liquid Biopsy

As exosomes have the potential to be used as prognostic biomarkers, isolation and identification of exosomes and their contents are also critical issues. For exosome isolation/purification, various methods have been evaluated. The most common method is differential centrifugation at 300× *g* for 10 min, 2000× *g* for 10 min, and 10,000× *g* for 30 min, followed by ultracentrifugation at 100,000× *g* for 2 h. For higher purity of exosomes, gradient centrifugation using sucrose can also be used [78]. An immuno-isolation method using antibody-coated magnetic beads can also be used to obtain higher purity and recovery rates. Rapid surface protein characterization using flow cytometry provides additional benefits with this method [79]. However, only specific types of exosomes can be isolated using this method, which can be considered as one of the limitations of this method. Currently, exosome extraction kits such as the ExoSpin^TM^ Exosome purification Kit (Cell Guidance Systems LLC; St. Louis, MO, USA) and Total Exosome Isolation Kit^TM^ (Life Technologies; Waltham, MA, USA) are also available. Typically, these kits use polymers such as polyethylene glycol with centrifugation to induce exosome sedimentation [80]. Microfluidic technology enables rapid and precise isolation/purification of exosomes with a very small volume of samples using a micro-electromechanical system [81]. Turbidimetry-enabled particle purification liquid chromatography based on the size exclusion principle also has been proven superior in purification of EVs in biofluids [82]. Using biosensors is another technique to determine exosomes with higher sensitivity and automated analysis [83]. For identification of exosome contents, general methods such as polymerase chain reaction (PCR), next-generation sequencing (NGS), and proteomics can be used.

#### 3.1.2. Applications of Exosomes as Diagnostic Biomarkers for Cancer

Liquid biopsy tools such as CTCs, ctDNA, and exosomes have advantages in non-invasive diagnosis and prognosis over traditional tissue biopsy strategies. Prognostic potential of CTCs has been already tested for monitoring epithelium-originating tumors in clinical trials [84]. However, CTCs shed from the primary tumor are found as only a few CTCs per mL of blood among millions of erythrocytes or leukocytes, and CTC enrichment techniques are needed for their detection [85]. Fragmented DNA shed from tumor cells may reflect the genetic signature of tumors [86]. Analysis of ctDNA in blood is challenging because there is a small fraction of ctDNA among cell-free DNAs from leukocytes in the blood sample [87]. In addition, the heterogeneity of tumor cells makes determining tumor-specific mutation in the ctDNA sample difficult.

Compared to the limited amounts of CTCs or ctDNA in the bloodstream, exosomes can be detected not only in blood but also in urine, cerebrospinal fluid, or lymphatic exudate [88,89]. Exosomes can be actively involved in cellular communication by delivering various signaling molecules. They serve as effective carriers, as the lipid bilayer can protect the contents and directly deliver them to the target cells. Exosome contents include nucleic acids, enzymes, and various signaling proteins. The contents can vary depending on the cells of origin. Therefore, the identification of exosomal contents can provide important clues regarding the cells of origin, which makes them ideal biomarkers for the diagnosis of diseases such as cancer, infection, metabolic, and neurodegenerative disorders [90,91].

**Figure 4:**
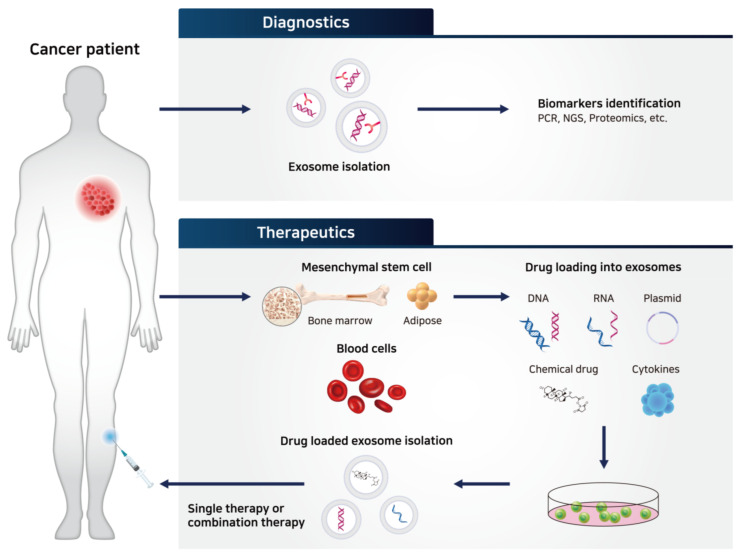
Potential therapeutic applications of exosomes in cancer. As exosomal components reflect the characteristics of the cells of origin, many attempts have been made to use tumor-derived exosomes (TEXs) as cancer diagnostic biomarkers. For identification of exosome contents, general methods such as polymerase chain reaction (PCR), next-generation sequencing (NGS), and proteomics can be used. Exosomes also have therapeutic potential as nanovehicles for drug delivery and personalized cancer immunotherapy. TEX: tumor-derived exosome; PCR: polymerase chain reaction; NGS: next-generation sequencing.

As exosomes and their contents released from cancer cells display unique properties, many attempts have been made to use TEXs as cancer diagnostic biomarkers (Table 1) [92]. TEXs play important roles in facilitating tumor growth and are involved in every step of cancer development, including angiogenesis, proliferation, metastasis, and fostering the tumor microenvironment by delivering relevant genes, growth factors, and cell signaling molecules [93,94,95]. For example, exosomes isolated from urine samples can be used to diagnose prostate cancer, bladder cancer, and glioblastoma. Typically, exosomal proteins related to epidermal growth factor receptor (EGFR) pathways (resistin, α-subunit of Gs protein, retinoic acid-induced protein 3, EGFR variant III, etc.) are present at diagnostic levels and hence, can be used as reliable biomarkers [96,97,98]. Prostate-specific antigen, survivin, and prostate cancer antigen 3 in exosomal contents can be also used for detecting prostate cancer [97,99,100]. Nucleic acids present in cancer exosomes, including miRNA, messenger RNA (mRNA), and long non-coding RNA (lncRNA), can also be used as diagnostic markers. For example, unique nucleic acids from exosomes have been identified in patients with glioblastoma [98]. Specific lncRNA, LINC00152, was also identified in gastric cancer-derived exosomes, which makes it a useful diagnostic biomarker [101]. miRNAs such as miR-21, -141, -200a, etc. can be detected in ovarian cancer patients, and miR-17-3p, -21, etc. were identified in lung cancer patients [102,103]. The genetic mutation in cancer patients is detectable by using exosome samples instead of CTCs or ctDNA. Exosomal RNA/DNA demonstrated the diagnostic value for KRAS mutation in pancreatic cancer and EGFR mutation in NSCLC [104,105,106]. BRAF mutation in EVs from lymphatic exudate of melanoma patients was reported to be useful for the prognosis [107]. Therefore, identification of unique exosomal contents corresponding to various types of cancers can help develop reliable diagnostic biomarkers (Figure 4).

### 3.2. Exosomes as Drug Delivery Vehicles for Oncotherapy

Exosomes are attractive nanovehicles for targeting cancer (Figure 4). As exosomes originate from endogenous cells, they possess low immunogenicity and thus induce low toxicity and side effects [108]. Exosomes are stable under physiological conditions. Owing to the presence of a lipid bilayer, they can protect the contents from the immune system and various enzymes. Furthermore, they demonstrate a homing capability by cell/tissue tropism with a longer circulation period, and can also cross the blood-brain barrier [109]. Unlike liposomes or other synthetic drug delivery nanoparticles, exosomes have characteristic membrane proteins and lipids that promote efficient targeting of exosomes to the recipient cell [110]. Exosomes can also enhance the delivery of contents as they can be directly fused or internalized into target cells. CD47, an integrin-associated protein upregulated in mesenchymal stem cells (MSCs), interacts with signal-regulatory protein, which helps inhibition of phagocytosis [111]. Thus, exosomes derived from fibroblast-like MSCs show the enhanced retention in the circulation in mice [6]. As the average size of exosomes ranges from 30 to 200 nm, passive targeting of exosomes to tumor tissue with enhanced permeability and retention effect can also be expected. With these benefits, a number of clinical and preclinical trials have been conducted to utilize exosomes as delivery vehicles.

#### 3.2.1. Methods for Loading Drugs into Exosomes

A number of studies have demonstrated that drug-loaded exosomes show better outcomes in inhibiting cancers, but the methods for loading drugs into exosomes also need to be explored further because they are closely related to the stability and loading efficiency of the drugs. To date, there are three types of drug loading methods for exosomes: exogenous loading, endogenous loading, and liposome fusion loading [108]. Exogenous loading refers to the method that directly entraps the drugs inside an isolated exosome with simple incubation, sonication, electroporation, repeated freeze/thaw, and extrusion [112]. Simple incubation can be easily used, but the average loading efficiency of paclitaxel into EVs was below 10%. Sonication can elevate the average loading efficiency up to 28.29%, but affecting the loading amount of hydrophobic drugs by altering the membrane of the exosome is an issue [113]. Typically, the exogenous loading method is advantageous in maintaining the aqueous stability of drug-loaded exosomes over one month at 4 °C and 37 °C, but it is limited by relatively low loading efficiency. Endogenous loading refers to a method that entraps desired molecules in exosomes by modifying the cells of origin before the isolation of exosomes. For instance, treating host cells with chemical drugs, such as paclitaxel, can induce the release of exosomes loaded with paclitaxel [114]. For protein or gene delivery, host cells can be transfected with desired genes, which facilitates the release of exosomes with desired proteins or genes [115]. Although the endogenous loading method demonstrates a relatively high loading efficiency, it is difficult to quantify the amount of content inside the exosome and maintain high purity. Exosomes used in membrane protein engineering approaches protect their cargo proteins, but can be degraded by proteinase [116]. The liposome fusion method uses the hybridization of drug-loaded liposomes and exosomes by the freeze/melting process. This fusion method exhibits higher loading efficiency, especially for loading large plasmids, including CRISPR-Cas9 expressing vectors [117]. However, it is unclear whether this hybridized liposome-exosome can maintain the unique properties of exosomes [118]. Hence, further comprehensive evaluation of parameters such as targetability, half-life, and side effects for hybridized liposome-exosome is required.

#### 3.2.2. Delivering Chemical Drugs via Exosomes for Oncotherapy

As many of chemical drugs can act after being internalized into cancer cells, they need to diffuse through the cell membrane to exert cytotoxicity, which is one of the factors reducing the efficacy of drugs [108]. In this aspect, exosomes can be potential candidates for delivering chemical drugs directly into the target cells. Many trials have been conducted to deliver chemical drugs such as paclitaxel, doxorubicin, cisplatin, and curcumin by packaging into exosomes for the treatment of various cancers (Table 2). For instance, paclitaxel-loaded exosomes isolated from MSCs, macrophages, and prostate cancer cells enhanced antitumor efficacy against pancreatic, breast, prostate, and Lewis lung carcinomas both in in vitro and in vivo studies [113,114,119,120]. Similarly, doxorubicin-loaded exosomes were also examined either by using the mechanical extrusion method to obtain higher drug loading efficiency [121] or surface engineering of exosomes to enhance targetability [122] for the treatment of colon and breast cancers, respectively. Treatment with cisplatin-loaded exosomes could prolong the survival rate of mice with ovarian cancer compared to the free cisplatin-treated group [123]. Pancreatic cancer-derived exosomes containing curcumin also effectively induced apoptosis in pancreatic cancer cells [124]. These studies show that by using exosomes as delivery vehicles, chemical drugs can be delivered more efficiently to target cells, which results in better outcomes.

#### 3.2.3. Delivering Therapeutic Proteins via Exosomes for Oncotherapy

As the efficacy of many proteins is limited due to several barriers such as short half-life, low delivery rate, and induction of resistance, the use of appropriate delivery vehicles is one of the best ways to achieve successful protein drug therapies. As exosomes can protect the contents from various enzymes and the immune system, they can act as effective delivery vehicles for proteins. Proteins can be loaded either inside or on the surface of the exosome, based on the mechanism of action of the drugs. However, as therapeutic proteins are macromolecules, it is difficult to directly incorporate them into exosomes. Therefore, genetic modification of the cells of origin leading to the expression of therapeutic proteins in exosomes is usually preferred to prepare protein-loaded exosomes. Several preclinical studies regarding protein-loaded exosomes are summarized in Table 2. For example, the delivery of tripartite motif-containing protein 3 (TRIM3) using gastric cancer-derived exosomes successfully suppressed the proliferation, migration, and metastasis of gastric cancer [125]. Similarly, apoptosis-inducing proteins such as suicide-inducing fusion protein or TNF-related apoptosis-inducing ligand (TRAIL) were loaded into exosomes, and this method could elicit substantially reduced tumor growth in in vivo tumor models [126,127]. Some signaling-related proteins can be expressed on the surface of exosomes to improve tumor immunity, such as the major histocompatibility (MHC) class I/peptide complex [128]. In other studies, immunogenic proteins such as HSP70 were loaded onto exosomes, which resulted in enhanced antitumor T cell activity [129]. Some studies have shown that EGFR nanobodies anchored on exosomes via glycosylphosphatidylinositol (GPI) could bind to EGFR-expressing tumor cells with higher affinity [130]. 

#### 3.2.4. Delivering RNA Drugs via Exosomes for Oncotherapy

Similar to therapeutic proteins, delivery vehicles are an essential component of successful gene therapy. Exosomes can protect genes from various enzymes, such as DNases and RNases, and can also directly deliver genes inside the cells, which enhances their therapeutic efficacy. Different types of RNAs such as mRNA, miRNA, and siRNA are promising candidates for the treatment of cancers. Studies on exosome RNA delivery are summarized in Table 2. miRNAs are non-coding RNAs involved in the regulation of gene expression. Pathophysiological conditions such as cancer are usually characterized by abnormal expression of certain types of miRNAs, which suggests that targeting miRNAs could be an effective way to treat cancer [144]. Treatment with exosomes overexpressing miR-122 showed substantially elevated chemosensitivity in hepatocellular carcinoma [134]. As the downregulation of miR-335-5p in both hepatocellular carcinoma and stellate cells acts as a pro-tumorigenic factor, delivery of miR-335-5p-overexpressing exosome exhibited substantial tumor shrinkage in an in vivo tumor model [135]. Similarly, treatment with miR-379-overexpressing exosomes significantly suppressed tumor growth in a T47D breast tumor model [136]. In addition, miR-145-5p overexpression inhibited the proliferation of pancreatic ductal adenocarcinoma and induced tumor cell apoptosis in an in vivo model [115]. In addition, specific miRNA inhibitors could act as potential drug candidates. For example, miR-25-3p is known to play an important role in facilitating colorectal cancer metastasis and promoting angiogenesis by targeting KLF (Kruppel-like factor)-2 and KLF-4, which implies that miR-25-3p can be a promising target for treating colorectal cancer. One study showed that treatment with exosomes loaded with miR-25-3p inhibitor considerably attenuated the tumor metastasis of colorectal cancer by balancing the level of miR-25-3p [137]. Silencing target genes using siRNA is another way to inhibit tumor growth. As overexpression of polo-like kinase (PLK)-1 is associated with the development of bladder cancer, one study showed that treatment with PLK-1 siRNA containing exosomes inhibited bladder cancer growth [138]. Similarly, as GRP78 overexpression is implicated in the growth and metastasis of hepatocellular carcinoma, treatment with GRP78 siRNA expressing exosomes resulted in an efficacious antitumor response in a sorafenib-resistant hepatocellular carcinoma model [139]. HSP27, a member of the heat-shock protein family, is known to promote neuron maturation and can be involved in the development of neuroblastoma. Treatment with Hsp27 siRNA-tagged exosome showed a significant reduction in tumor growth of the neuroblastoma cell line SH-SY5Y [140]. mRNA can be another candidate for anticancer therapy using exosome vehicles. Transferring CRISPR-associated protein (Cas) 9 mRNA-expressing exosomes from red blood cells induced miRNA inhibition and Cas9 genome editing effects in a breast cancer model [141]. Phosphatase and tensin homolog (PTEN) and esophageal cancer-related gene (ECRG) 4 are classified as tumor suppressors and are generally mutated in cancer cells. Therefore, treatment with exosomes expressing PTEN or ECRG4 mRNA could inhibit the growth of glioma cells [145] and tongue squamous cell carcinoma cells, respectively [143]. These studies show that depending on the target, different types of RNA therapeutics can be chosen, and the therapeutic efficacy of these drugs can be substantially enhanced by using exosomes as delivery vehicles.

### 3.3. Exosomes into Personalized Cancer Immunotherapy Drug Design (Single or in Combination)

Exosomes can be used as cell-free vaccines owing to the fact that exosomes derived from various donor cells, such as immune cells and cancer cells, are involved in fostering antitumor immunity [146]. DEXs can perform important immunostimulatory functions, as DCs that act as sentinel antigen-presenting cells play a crucial role in orchestrating cancer-specific adaptive immunity [147]. The surface of DEXs is characterized by various functional molecules for priming T cells such as MHC class I/II and costimulatory molecules including CD40, CD80, and CD86 [148]. This can foster antitumor immunity by inducing the activation of both innate and adaptive immunity. In several preclinical tests, treating DEXs could elicit antitumor effects and prolong the survival rate of tumor-bearing mice by expanding the repertoire of tumor-specific cytotoxic T cells as well as activating naïve T cells [149]. DEXs are also known to induce NK cell-mediated cytotoxicity to inhibit tumor growth [150]. In order to potentiate the efficacy of DEX as a therapeutic cancer vaccine, choosing an appropriate TAA and a relevant adjuvant is essential. To date, both for human and preclinical studies, only MHC class I/II binding peptides such as Epstein-Barr virus, melanoma-associated antigen, and melanoma antigen recognized by T cells-1 have been used for DEX vaccine [13,14,151]. However, the use of peptide-based DEX vaccine was not very effective in inducing antitumor effects due to modest activation of antitumor immune responses [152]. Several studies have reported that a more intense adaptive immune response can be induced with a DEX vaccine loaded with protein antigen [152,153]. This effect might be attributable to the presence of a broad range of epitopes with protein antigens, which might be more effective in activating various repertoires of tumor-specific cytotoxic T cells. These studies demonstrate the ability of personalized DEX vaccines based on patient tumor lysates. Therefore, a more potent DEX vaccine that can evoke strong antigen-specific responses can be manufactured with the loading of an allogenic protein antigen. Based on other studies, B cells are required to boost antitumor immunity, suggesting that epitopes, which activate B cells, are also needed for a successful DEX vaccine [152,153]. These results also provide a rationale for utilizing personalized protein antigens as cargo in DEX vaccines. General adjuvants such as interferon-γ and toll-like receptor agonists, including polyinosinic:polycytidylic acid and CpG oligodeoxynucleotides can be used to potentiate the efficacy of DEX vaccine. It is also known that the use of these adjuvants can result in the maturation of DCs and ultimately produce more immunogenic DEXs [154]. DEXs derived from mature DCs are known to express more costimulatory surface molecules including CD40, CD80, CD86, and intercellular adhesion molecule -1 and MHC class I/II [149]. Even though the DEX vaccine seems to show promising outcomes, several challenges remain until it can be widely used in clinics, as the research on this therapy is still at an early stage. For example, it is not clear whether the mass production of personalized DEX vaccine possessing a homogenous quality is available. At present, there are no clear guidelines regarding the production of exosome-based therapeutics. Proper storage, maintenance of stability, and route of administration for exosomes are also the issues to be considered [155]. 

Besides, CAR exosomes derived from effector CAR-T cells showed cytotoxic effects on cancer cells [156]. Intravenous injection of CAR exosomes into a mouse xenograft model exerted potent tumor growth inhibition. A combination of exosomal therapy with other immunotherapies such as immune-checkpoint blockers, cytokines, adoptive T cell transfer, and cancer vaccines might be a good way to elicit synergistic anticancer effects. For the combined utilization of CAR exosomes and CAR-T cells, further clinical/preclinical studies are required, and the clinically applicable scheme should be proposed.

## 4. Conclusions and Future Perspectives

Recently, cancer therapeutics has made great strides, and various clinical trials for targeted cancer therapy or immunotherapy have been conducted singly or in combination. However, there remains an unmet need, because only a few types of cancer patients are restrictedly responsive to current immune checkpoint blockers. In order to elevate response rate for personalized immunotherapy, prognostic biomarkers need to be established. As mentioned above, exosomal components can be used as diagnostic biomarkers in liquid biopsy, nanovehicles for delivery of anticancer drugs, and mediators between cells affecting tumor immunity. Detection of marker proteins or nucleic acids in circulating exosomes shows potential for predicting patients’ clinical response [157]. “TEXs-on-chip” techniques using patient-derived tumor spheroids obtained from liquid biopsy will be applicable in the near future. 

In that vaccinating autologous exosomes obtained from patients can avoid allograft reaction and carry tumor antigens, patient-derived exosomes have received attention as good candidates for personalized immunotherapy. Using bone marrow aspirate from patients, MSCs are isolated and expanded ex vivo, and then large scale of MSCs engineered with anticancer genes can be transplanted to patients for personalized treatment [158]. As exosomes released from MSCs acquire tropism toward tumor locations and the corresponding receptors with the original MSC, they would mediate anticancer activity [159]. Engineering techniques for enhancing therapeutic efficacy of these cell-free vaccines are needed for the development of innovative personalized immunotherapy. 

## Figures and Tables

**Table 1:** Applications of exosomes as diagnostic biomarkers for cancer.

Exosome Contents	Associated Molecule	Target Disease	Reference
**Proteins**	Resistin, α-subunit of Gs protein, retinoic acid-induced protein 3	Prostate, bladder cancer	[96,97]
	Epidermal growth factor receptor (EGFR) variant III	Glioblastoma	[98]
	Prostate-specific antigen, survivin, prostate cancer antigen 3	Prostate cancer	[97,99,100]
**RNAs**	lncRNA, LINC00152	Gastric cancer	[101]
	miR-21, miR-141, miR-200a	Ovarian cancer	[102]
	miR-17-3p, miR-21	Non-small cell lung cancer (NSCLC)	[103]
**DNAs**	Mutant KRAS	Pancreatic cancer	[104]
	EGFR T790M mutation	NSCLC	[105]
	Mutant KRAS, TP53	Pancreatic cancer	[106]
	BRAF^V600E^ mutation	Melanoma	[107]

EGFR: epidermal growth factor receptor; NSCLC: non-small cell lung cancer.

**Table 2:** Exosomes as drug delivery vehicles for oncotherapy in preclinical studies.

Therapeutic Molecules	Exosome Origin	Targeted Disease	Reference
**Chemical drugs**			
Paclitaxel	Macrophage	Lewis lung carcinoma	[113]
	MSC	Pancreatic, breast cancer	[114,119]
	Prostate cancer cell	Prostate cancer	[120]
Droxorubicin	U937 RAW264.7	Colon cancer	[121]
	DCs expressing iRGD	Breast cancer	[122]
Cisplatin	Hepatocarcinoma cell	Hepatocarcinoma	[123]
Curcumin	Pancreatic cancer cell	Pancreatic cancer	[124]
**Proteins**			
TRIM3	Gastric cancer cell	Gastric cancer	[125]
CD-UPRT fusion protein	HEK293T	Schwannoma	[126]
TRAIL	K562	Lymphoma	[127]
MHC class I/peptide complex	DC	Breast cancer	[128]
HSP70	Myeloma cell	Myeloma	[129]
EGFR nanobodies	Myeloid leukemia cell	Epidermal carcinoma	[130]
SIRPα	Embryonic kidney cell	Colon cancer	[131]
**miRNA**			
miR-145-5p	MSC	Pancreatic cancer	[115]
Let-7a	HEK293T expressing GE11	Breast cancer with EGFR	[132]
miR-146b	MSC	Glioma	[133]
miR-122	MSC	Hepatocellular carcinoma	[134]
miR-335-5p	Stellate cell	Hepatocellular carcinoma	[135]
miR-379	MSC	Breast cancer	[136]
miR-25-3p inhibitor	Colorectal cancer cell	Colorectal cancer	[137]
**siRNA**			
PLK-1 siRNA	HEK293T + MSC	Bladder cancer	[138]
GRP78 siRNA	MSC	Hepatocellular carcinoma	[139]
HSP27 siRNA	Neuroblastoma cell	Neuroblastoma	[140]
**mRNA**			
Cas9 mRNA	Red blood cell	Breast cancer	[141]
PTEN mRNA	Mouse embryonic fibroblast serum	Glioma	[142]
ECRG4 mRNA	Neuroblastoma cell	Tongue carcinoma	[143]

MSC: mesenchymal stem cell; DC: dendritic cell; TRIM: tripartite motif-containing protein; CD: cytosine deaminase; UPRT: uracil phosphoribosyltransferase; TRAIL: TNF-related apoptosis-inducing ligand; MHC: major histocompatibility complex; HSP: heat shock protein; EGFR: epidermal growth factor receptor; SIRP: signal-regulatory protein; PLK: polo-like kinase; Cas: CRISPR associated protein; PTEN: phosphatase and tensin homolog; ECRG: esophageal cancer related gene.

## Abbreviations

ADC	Antibody-drug conjugate
ALIX	Apoptosis-linked gene 2-interacting protein X
ALK	Anaplastic lymphoma kinase
CAF	Cancer-associated fibroblasts
CAR	Chimeric antigen receptor
Cas	CRISPR-associated protein
CD	Cytosine deaminase
CSF1R	Colony-stimulating factor-1 receptor
CTC	Circulating-tumor cell
ctDNA	Circulating tumor DNA
CTLA4	Cytotoxic T-lymphocyte-associated protein 4
DC	Dendritic cell
DEX	Dendritic cell-derived exosome
ECM	Extracellular matrix
ECRG	Esophageal cancer-related gene
EGFR	Epidermal growth factor receptor
EpCAM	Epithelial cell adhesion molecule
ER	Endoplasmic reticulum
ESCRT	Endosomal sorting complex required for transport
EV	Extracellular vesicle
FGFR	Fibroblast growth factor receptor
FLT3	FMS-like tyrosine kinase 3
GM-CSF	Granulocyte-macrophage colony-stimulating factor
GPI	Glycosylphosphatidylinositol
HRS	Hepatocyte growth factor-regulated tyrosine kinase substrate
HSP	Heat shock protein
ICAM	Intercellular adhesion molecule
ILV	Intraluminal vesicle
KLF	Kruppel-like factor
lncRNA	Long non-coding RNA
MHC	Major histocompatibility complex
miRNA	MicroRNA
MMP	Matrix metalloproteinase
mRNA	Messenger RNA
MSC	Mesenchymal stem cell
MV	Microvesicle
MVB	Multivesicular body
NGS	Next-generation sequencing
NK	Natural killer
NSCLC	Non-small cell lung cancer
NTRK	Neurotrophic tyrosine receptor kinase
PCR	Polymerase chain reaction
PD-1	Programmed cell death protein 1
PD-L1	Programmed cell death ligand 1
PLK	Polo-like kinase
PTEN	Phosphatase and tensin homolog
ROS1	C-ros oncogene 1
RTK	Receptor tyrosine kinase
siRNA	Small interfering RNA
SIRP	Signal-regulatory protein
TAA	Tumor-associated antigen
TEX	Tumor-derived exosome
TMZ	Temozolomide
TRAIL	TNF-related apoptosis-inducing ligand
TRIM	Tripartite motif-containing protein
UPRT	Uracil phosphoribosyltransferase
